# The Effects of Ester and Ether Polycarboxylate Superplasticizers on the Fluidity and Setting Behavior of Alkali-Activated Slag Paste

**DOI:** 10.3390/ma17204951

**Published:** 2024-10-10

**Authors:** Yong Jic Kim, Sung Choi, Sung Rok Oh

**Affiliations:** 1 Division of Smart Construction and Environmental Engineering, Daejin University, Pocheon 11159, Republic of Korea; yong1yong2@hotmail.com; 2 Department of Civil Engineering, Kyungdong University, Yangju 11458, Republic of Korea; 3 R&D Team, Newjust Co., Ltd., Gwangmyeong 14348, Republic of Korea

**Keywords:** alkali-activated slag, ester polycarboxylate superplasticizer, ether polycarboxylate superplasticizer, fluidity, setting time

## Abstract

This study aims to investigate the comparative performance of ester- and ether-based polycarboxylate superplasticizers in maintaining the fluidity and controlling the setting time of alkali-activated slag (AAS) paste. The experiments employed rheological tests, mini-slump tests, ultrasonic pulse velocity (UPV) measurements, and gel permeation chromatography (GPC) analysis. The results indicate that ether-based superplasticizers maintain fluidity approximately 25% longer than their ester-based counterparts and extend the setting time by about 30%. The enhanced performance of ether-based superplasticizers is attributed to their superior molecular stability in highly alkaline environments, which mitigates early polymer degradation. Additionally, the Na_2_O/SiO_2_ ratio was maintained at 1:1 throughout the experiments to ensure consistency in the activation process. The relationship between fluidity loss and the onset of setting occurs more rapidly in AAS paste than in conventional cement-based systems. These findings provide valuable insights for the development of environmentally friendly construction materials by optimizing the use of superplasticizers in alkali-activated systems.

## 1. Introduction

Concrete has long been used as a primary construction material for various infrastructures, with Portland cement being the main binder due to its excellent engineering properties, low cost, and ease of mass production. However, the environmental impact caused by carbon emissions during cement production has necessitated the development of eco-friendly alternatives [1]. Cement production is responsible for approximately 7–8% of global CO_2_ emissions [2], which has spurred interest in sustainable construction materials [3].

Alkali-activated slag (AAS) concrete is a promising alternative that can deliver similar physical performance to Portland cement-based concrete without the use of conventional cement. AAS concrete is produced by activating ground granulated blast furnace slag (GGBFS) with alkali activators [4]. In addition to exhibiting superior durability and chemical resistance compared to ordinary Portland cement (OPC)-based concrete, AAS concrete can achieve high early strength, reducing formwork retention times [5,6]. Furthermore, since GGBFS is an industrial byproduct, AAS concrete offers environmental benefits by minimizing CO_2_ emissions associated with OPC [7,8].

Despite these advantages, one of the major challenges in the practical application of AAS concrete is the rapid loss of fluidity and fast setting time, potentially limiting workability [9,10]. The quick stimulation of the GGBFS reaction by alkali activators leads to a high initial hardening rate, potentially leading to shrinkage cracks before the concrete has developed sufficient strength [11]. Therefore, modifying the initial fluidity and delaying the setting time are crucial for the wider adoption of AAS concrete [12,13].

Polycarboxylate superplasticizers (PCEs) are widely used to enhance the fluidity of concrete, and they can be classified into ester (ES) and ether (ET) types [14]. Previous research has shown that naphthalene-based superplasticizers can enhance the fluidity of AAS in highly alkaline environments but may not maintain fluidity over extended periods [15]. PCEs, on the other hand, have been shown to have better potential for maintaining fluidity in such environments [16,17].

Research on the molecular stability of superplasticizers in highly alkaline AAS environments has shown that ester-based superplasticizers tend to degrade under these conditions, while ether-based superplasticizers more effectively retain their molecular structure [18,19,20]. Rheological characteristics of AAS paste are significantly influenced by the type of superplasticizer used, with ether-based polycarboxylates maintaining fluidity longer than their ester-based counterparts [21,22]. Additionally, studies using ultrasonic pulse velocity (UPV) and other measurement techniques have shown that the viscosity measurement limit (VML) can be used to quantitatively assess the setting process in AAS systems [23,24].

Recent studies have also focused on the molecular structure and adsorption mechanisms of PCEs in alkali-activated systems. Mohamed et al. (2022) explored the molecular modeling of PCEs and highlighted their potential limitations in highly alkaline environments [25,26,27]. Liu et al. (2022) examined the interaction mechanisms between PCEs and cement particles, emphasizing the influence of functional groups on the superplasticizer’s performance in maintaining fluidity [28]. In addition, recent experimental work by Fan et al. (2023) and Xun et al. (2020) has provided insights into the rheological properties and mechanical strengths influenced by various types of superplasticizers in AAS systems [29,30].

This study aims to investigate the effects of ester- and ether-based PCEs on the initial fluidity and setting characteristics of AAS paste. Specifically, we analyze the molecular structure stability of these superplasticizers in an alkaline environment and quantitatively assess the relationship between fluidity loss and setting time using rheological tests and other measurements. This research provides insights into optimizing the use of PCEs in AAS systems to improve fluidity retention and adjust setting times for more practical applications in eco-friendly construction materials.

## 2. Materials and Methods

### 2.1. Materials and Mixture Proportions of AAM Mortar

**Figure 1:**
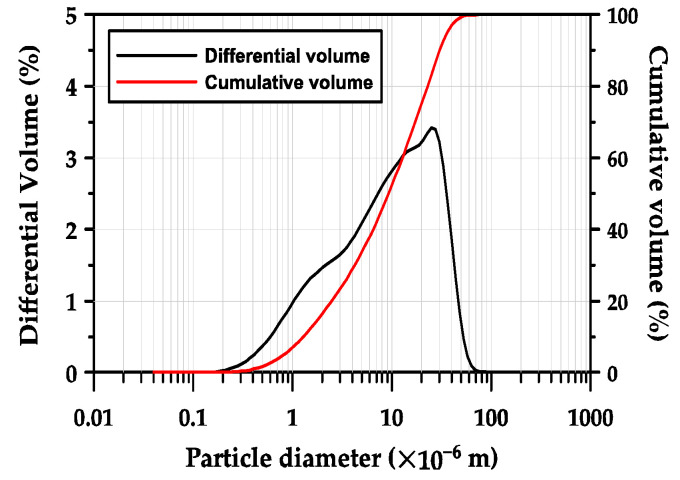
Particle size distribution of GGBFS.

GGBFS obtained from POSCO (Pohang, in the Republic of Korea) was used as the primary binder. The chemical composition of the GGBFS, determined using X-ray fluorescence (XRF, Bruker S8 Tiger, in Billerica, MA, USA) is presented in Table 1. The GGBFS was composed of 44.0% CaO, 33.7% SiO_2_, 13.8% Al_2_O_3_, and 5.2% MgO. Thus, its basicity coefficient (Kb = (CaO + MgO)/(SiO_2_ + Al_2_O_3_)) was 1.04, which is similar to the neutral value of 1.0 preferred for alkali activation [31]. The hydraulic modulus (HM = (CaO + MgO + Al_2_O_3_)/SiO_2_) was 1.87, which was 33.5% higher than the required value of 1.4 for good hydration properties of GGBFS [23,31]. Figure 1 shows the particle size distribution of GGBFS measured using a laser particle size analyzer (PSA, Beckman Coulter LS 13 320, Brea, CA, USA). The mean particle size (d50), which represents the particle size of a cluster of particles, was 10.2 μm, and the d10 and d90 values were 1.4 μm and 33.2 μm, respectively. 

Two types of alkaline activators, namely, sodium hydroxide (98% purity) and sodium silicate (type 3 industrial water glass, Na_2_SiO_3_), were used to activate the GGBFS. The sodium silicate solution had a chemical composition of 28.3 wt% SiO_2_, 9.3 wt% Na_2_O, and 62.4 wt% H_2_O. A 4 M sodium hydroxide solution was prepared by mixing it with deionized water. The alkaline activator was stored in a chamber at 20 °C and a relative humidity (RH) of 60% for 24 h prior to casting so that it cooled down to ambient temperature (NaOH releases heat when mixed with water). The pH of the activator was 14.

The PCEs used in AAS pastes were two ester-based PCEs (ES1 and ES2) and two ether-based PCEs (ET1 and ET2) produced by Company K (UlSan-si, Republic of Korea). Table 2 lists their basic properties.

Table 3 shows the mixture design of the AAS pastes used for the evaluation of fluidity and setting. In this study, a liquid-to-binder ratio (l/b, where the binder content is defined as the GGBFS content) was fixed at 0.42. The content of Na_2_O was 6% of the binder mass, and the SiO_2_/Na_2_O molar ratio was maintained at 1.0, which has been identified as the optimal ratio for alkali activation and stability during the reaction in previous studies [32]. The content of the PCEs was 1.0% by mass of the binder.

### 2.2. Test Methods

#### 2.2.1. Gel Permeation Chromatography (GPC)

PCEs are polymer materials, but their molecular structures can be changed in an alkaline environment owing to the separation of side chains and main chains. The GPC test was conducted to examine the changes in the molecular weight distribution of polycarboxylate superplasticizers. The GPC equipment used was an Agilent 1100s (Santa Clara, CA, USA). GPC is a type of liquid chromatography of the liquid–solid phase through the elution method. Regarding the measurement principle, when polymers move along a fluidized bed, a sample with a large molecular weight requires a shorter time to pass through the column, whereas a sample with a small molecular weight requires a longer time depending on the size of the chain. The molecular weight of polymers can be measured by comparing this time with the calibration curve of the standard sample. It is possible to understand and predict the properties of polymers through the GPC test, and the test is used to determine the molecular weight distribution and relative average molecular weight. The purpose of this test was to assess how the alkaline environment affected the molecular structure and stability of the polycarboxylate superplasticizers, particularly in terms of chain separation and degradation.

#### 2.2.2. Heat of Hydration

A micro heat of hydration test was conducted to evaluate the hydration reaction of the AAS paste with polycarboxylate superplasticizers. A three-point multipurpose condition calorimeter manufactured by Tokyo Riko Co. Ltd. (Tokyo, Japan) was used. The temperature of the equipment was varied over the range of 5–60 °C, and the temperature stability was 5 × 10^−3^ °C/5 °C. The equipment was initially set at 20 °C. The GGBFS, alkali activators, water, and PCEs were placed in a vial and inserted into the calorimeter, then they were mixed for five minutes using an internal propeller. The heat of hydration was measured every five minutes for one hour, and the temperature increase was recorded for analysis. The purpose of this test was to investigate how the PCEs influenced the hydration kinetics of AAS paste and to assess their impact on the heat generation during the hydration process.

#### 2.2.3. Fluidity

The fluidity of the AAS pastes was evaluated using the mini-slump test to assess the initial workability of the mixtures with PCEs. The test was conducted in accordance with ASTM C1437-15 [33]. The mold for slump measurement was made of brass. The cylindrical mold had an upper inner diameter of 70 mm, a lower inner diameter of 100 mm, and a height of 50 mm. The mini-slump was measured every 15 min from 0 to 60 min immediately after mixing. Before the measurement, remixing was performed for 30 s to exclude the influence of false set and any moisture evaporation from the AAS paste surface. The purpose of this test was to determine how the polycarboxylate superplasticizers influenced the fluidity over time and to evaluate their effectiveness in maintaining the workability of the AAS pastes.

#### 2.2.4. Setting Time

Both the Vicat (per ASTM C191-18A [34]) and rheology/UPV tests were conducted to measure the setting time of the AAS pastes. The elapsed time between the initial contact of cement and water and the penetration of 25 mm is defined as the Vicat initial time of setting. The Vicat final time of setting was determined when the first penetration measurement did not mark the specimen surface with a complete circular impression. The purpose of this test was to determine the initial and final setting times of AAS pastes and to evaluate how the PCEs influenced the setting behavior of the material.

#### 2.2.5. Rheology/UPV

A paste viscometer manufactured by Brookfield was used to evaluate the rheological characteristics of the AAS paste [35,36]. Table 4 shows its specifications. The viscometer measures the plastic viscosity and yield stress using the shear stress generated while the spindle rotates in the paste. The test method is divided into variable rate rheology (VRR) and controlled constant rheology (CCR) tests according to the spindle speed of the viscometer. The viscosity of the AAS paste was measured using both test methods. In the VRR test, the spindle was inserted into the AAS paste in the fluid state, and the shear stress generated in the spindle was measured according to the rotation speed (5 to 100 rpm). From the test results, the plastic viscosity and yield stress were calculated through the regression of the shear stress according to the rotation speed, based on the theory of Bingham fluids. In the VRR test, measurements were performed every 15 min from 0 to 60 min, and remixing was performed for 30 s before placing the paste in the measuring equipment, as with the mini-slump test. In the CCR test, the spindle was inserted into the paste, and the shear stress generated by the spindle was measured while it rotated at a rate of 5 rpm. The shear stress of the fluid state was measured using the spindle until it no longer provided a measurement. All the rheology tests were conducted in a chamber at a constant temperature and humidity of 23 ± 0.5 °C and 60 ± 5% RH, respectively. The purpose of the rheology tests was to evaluate how the PCEs influenced the viscosity and yield stress of AAS pastes over time, providing insights into their effect on the workability and flow behavior of the material.

The UPV test was performed by placing the oscillator and receiver on acrylic boxes (thickness: 10 mm; ultrasonic velocity of acrylic: 2740 m/s) placed 20 mm apart, filling the container with paste, and measuring the transmission time of ultrasonic waves between the oscillator and receiver at intervals of 30 s at a measurement frequency of 5 kHz. The purpose of the UPV test was to assess the setting process by measuring the propagation speed of ultrasonic waves, which provides information on the internal structure development of the AAS paste.

## 3. Results and Discussion

### 3.1. GPC

PCEs, which are polymer synthetic compounds in the liquid state, consist of main chains and side chains. In a strongly alkaline environment, the dispersion function of the admixtures is lost because the connection between the main chains and side chains is destroyed [37]. The GPC test was conducted to examine the molecular structure of polycarboxylate superplasticizers according to the pH condition, comparing neutral (pH 7.0) and strongly alkaline (pH 13.4) environments. The alkaline condition was achieved using alkali activators, such as NaOH and Na_2_SiO_3_, commonly used in AAS pastes.

**Figure 2:**
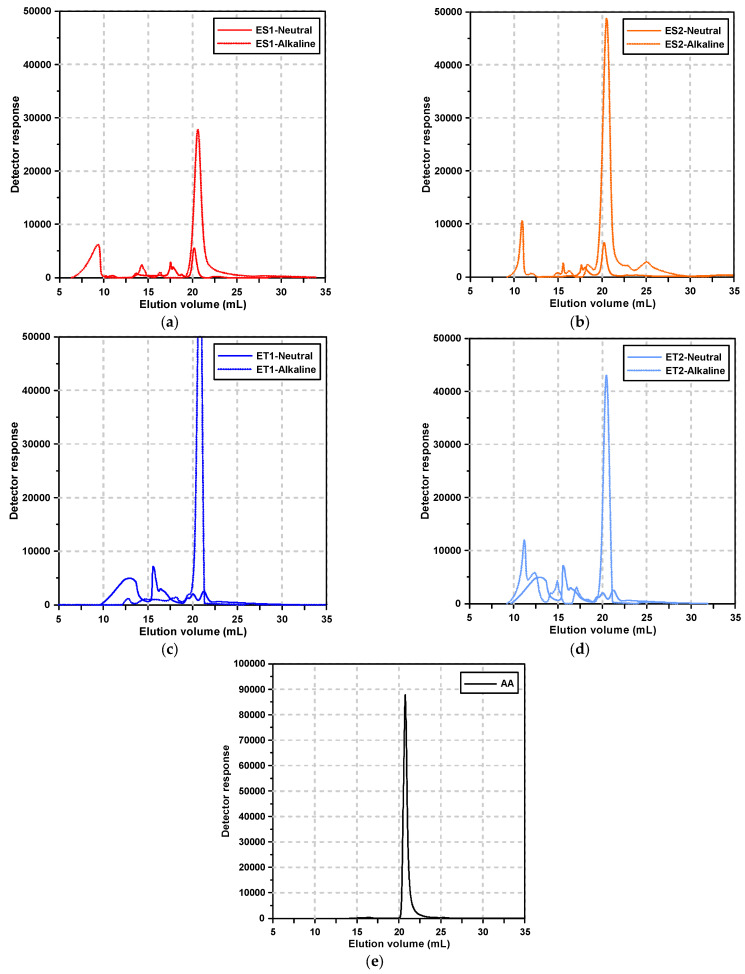
Results of the GPC test: (**a**) ES1, (**b**) ES2, (**c**) ET1, (**d**) ET2, and (**e**) alkali activator.

Figure 2 shows the GPC elution curves of the polycarboxylate superplasticizers and alkali activator according to the pH condition. Figure 2e, specifically, depicts the GPC results of the alkali activator solution, which exhibited a sharp, single peak at an average elution volume corresponding to 9.62 g/mol. This peak is indicative of the small molecular components in the alkali activator.

Under neutral conditions, the polycarboxylate superplasticizers exhibited multiple elution peaks, reflecting the presence of various molecular weight distributions due to the synthesis process. These multiple peaks are typical for polymeric materials, as they often contain components of varying molecular weights due to polymerization kinetics and the presence of residual monomers or by-products.

However, under alkaline conditions, significant changes were observed in the elution profiles of polycarboxylate superplasticizers. Notably, for the ester-based polycarboxylate superplasticizers, the range of elution volumes broadened, and new peaks corresponding to smaller molecular sizes appeared. This suggests that the molecular degradation of the polymer occurred in the strongly alkaline environment, particularly involving the separation of side chains from the main chains.

The ester-based superplasticizers, synthesized using methacrylic acid (MAA) and methoxy polyethylene glycol (MPEG), are known to have ester bonds that are susceptible to hydrolysis in alkaline conditions. The hydrolysis of these ester bonds results in the cleavage of MPEG side chains from the main MAA backbone, leading to the formation of smaller molecular fragments, as evidenced by the appearance of new peaks at lower elution volumes.

This molecular breakdown highlights the vulnerability of ester bonds to nucleophilic attack by hydroxide ions (OH^−^) in the alkaline environment, a common mechanism in ester hydrolysis. The separation of the MPEG side chains from the MAA backbone disrupts the polymer’s ability to provide effective dispersion, resulting in the loss of its superplasticizing function.

In contrast, the ether-based polycarboxylate superplasticizers showed less variation in their elution volume range under alkaline conditions. The absence of new peaks in the lower molecular weight region suggests that the ether bonds in these polymers are more resistant to hydrolytic degradation. Ether-based superplasticizers are synthesized by polymerizing allyloxy-polyethylene glycol (APEG) with acrylic acid (AA). The ether group (-C-O-C-) is known for its chemical stability, particularly in alkaline environments. This stability is attributed to the strength of the ether bond, which is less prone to nucleophilic attack compared to ester bonds.

However, subtle shifts in the elution peaks of the ether-based superplasticizers under alkaline conditions indicate that, while the polymer did not undergo significant chain cleavage, some structural rearrangements or molecular interactions likely occurred. These changes could be attributed to secondary interactions, such as the reconfiguration of polymer chains or interactions with the surrounding alkaline environment, rather than actual degradation. This suggests that, although ether-based superplasticizers retain their structural integrity in alkaline conditions, their performance may still be affected by minor conformational changes or interactions with other components in the AAS paste.

### 3.2. Heat of Hydration

The reaction rate and heat release of the AAS paste vary depending on the activator type and content, and two to three hydration heat peaks generally occur at early ages. The first hydration heat peak is relatively small due to the initial reaction that breaks down the surface structure of the slag, releasing ions. This initial surface reaction occurs rapidly but does not contribute significantly to the overall heat release.

The second hydration heat peak, however, is generated as the ions are further eluted from the interior of the slag, initiating a more substantial hydration reaction. As a result, the second peak is typically much higher than the first, reflecting the onset of more extensive hydration reactions. When the content of alkali activators is high, the second peak occurs earlier, which may cause overlap between the first and second hydration heat peaks. In such cases, the distinct separation of the two peaks may diminish, leading to a combined heat release profile. When two or more alkali activators are used, distinct hydration heat peaks reflect the characteristics of each activator component [38].

**Figure 3:**
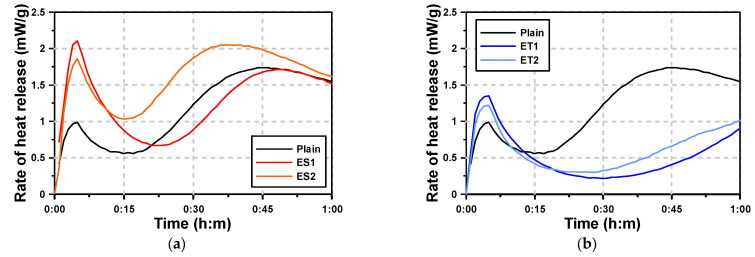
Results of heat of hydration with chemical admixtures: (**a**) ES and (**b**) ET.

Figure 3 shows the microhydration heat test results of the AAS paste with the polycarboxylate superplasticizers. For Plain AAS paste, which used NaOH and Na_2_SiO_3_ as activators, two hydration heat peaks were observed within the first hour. The first peak occurred at approximately 7 min, and the second peak at approximately 45 min. The first peak is associated with the rapid dissolution of NaOH, while the second peak is attributed to the slower reaction of Na_2_SiO_3_. While the paste activated with NaOH had a low early hydration heat and no clear induction period, the paste activated with Na_2_SiO_3_ exhibited a large second hydration heat peak after the occurrence of the initial peak. This indicates that Na_2_SiO_3_ plays a dominant role in determining the overall heat release pattern, especially in contributing to the second peak. Plain was significantly affected by the hydration heat pattern of Na_2_SiO_3_, and the second peak was larger than the first peak.

The one-hour hydration heat curves of the AAS pastes with the polycarboxylate superplasticizers (ES and ET) in Figure 3b show that the first hydration heat peak occurred at similar times for all samples. Especially when ES was used, the first peak was observed to be large. This suggests that the presence of ES facilitated a more uniform dispersion of the AAS binder, allowing for an enhanced early reaction. For ES, the second hydration heat peak occurred at similar times to those of Plain. In the case of ET, the first hydration heat peak was higher than that of Plain, but lower than that of ES. However, the second hydration heat peak was not observed within the first hour, indicating a delayed hydration process. Instead, an inflection point, where the hydration heat began to increase again, was observed. This suggests that ET delayed the onset of the second major hydration reaction, possibly due to its impact on the diffusion of ions from the slag particles. This is because the dissolved ions from the slag underwent a hydration reaction at the point where the hydration heat rose again (second hydration heat peak) after the first peak.

Table 5 presents the time of the second hydration heat inflection point and VML. It is evident that the timing of the second hydration heat inflection point aligns closely with the VML (viscosity measurement limit), marking the point where the AAS paste begins to lose its plastic viscosity. The results show that the time at which the second hydration heat inflection point occurred was similar to the VML, at which point the AAS paste lost its plastic viscosity. This alignment suggests a close relationship between the loss of plasticity and the onset of the second hydration phase. In particular, the timing tended to coincide for Plain and ES pastes, indicating that the hydration process and viscosity loss occurred almost simultaneously in these samples. This is because the second hydration heat peak rapidly increased for Plain and the AAS paste with ES. However, in the case of ET, there was a difference of approximately ten minutes between the second hydration heat inflection point and the VML. The delayed reaction in ET indicates that the formation of hydrates, which leads to hardening, occurred more gradually compared to Plain. This is because the hardening action caused by the formation of hydrates was delayed for ET, as the second hydration heat peak increased more slowly compared to that of Plain.

### 3.3. Fluidity

**Figure 4:**
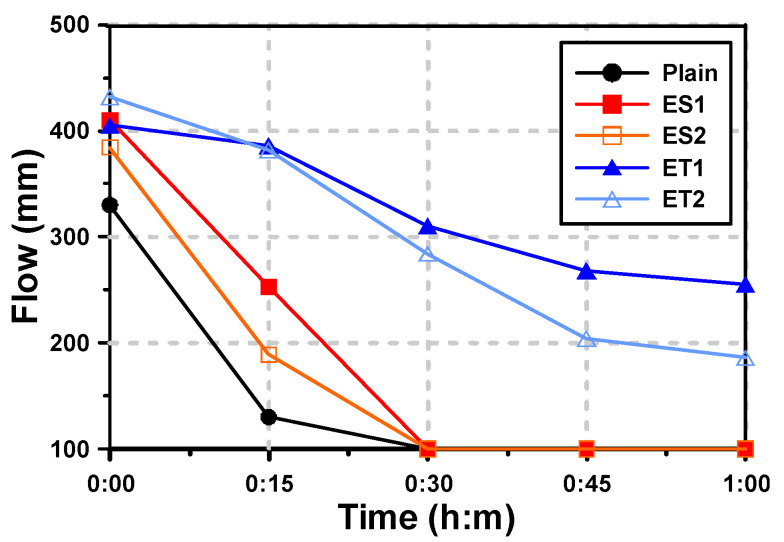
Results of flow for AAS pastes with chemical admixtures.

Figure 4 shows the mini-slump test results of the AAS pastes with both the ether- and ester-based polycarboxylate superplasticizers. For the paste denoted “Plain,” which had no polycarboxylate superplasticizer, the initial fluidity (mini-slump flow) was 330 mm, and the fluidity rapidly decreased after 15 min. After 30 min, the fluidity was entirely lost. This rapid loss of fluidity in the Plain sample is likely due to the high reactivity of alkali-activated materials, where the initial setting and hardening occur quickly without the use of superplasticizers to delay the process.

It has been reported that the use of polycarboxylate superplasticizers further improves the initial fluidity of AAS mortar [19]. In the results of this study, the use of polycarboxylate superplasticizers also increased the initial fluidity. The improvement in initial fluidity can be attributed to the superplasticizers’ ability to disperse the binder particles more effectively, reducing the water demand and enhancing the free water content available for flow. The fluidity of ES1 and ES2, which are ester-based polycarboxylate superplasticizers, increased by 24.2% (ES1) and 16.4% (ES2) compared with that of Plain. The fluidity of ET1 and ET2, which are ether-based polycarboxylate superplasticizers, increased by 22.7% (ET1) and 30.9% (ET2) compared with that of Plain. This indicates that both ether- and ester-based polycarboxylate superplasticizers improve the dispersion of the slag particles, but the extent of the improvement varies between the two types. However, the tendency for decreased fluidity over time was different depending on the type of polycarboxylate superplasticizers used.

The fluidity of the ester-based polycarboxylate superplasticizers rapidly decreased over time, and was completely lost after 30 min. The Plain mixture also showed a similar phenomenon. Both Plain and ester-based polycarboxylate superplasticizers suddenly hardened within tens of seconds. This rapid loss of fluidity is likely due to the hydrolysis of ester bonds in the polycarboxylate structure under alkaline conditions, leading to a breakdown of the polymer chains and a reduction in dispersion efficiency.

The fluidity of the ether-based polycarboxylate superplasticizers also decreased over 60 min after paste mixing, but it was not completely lost. The fluidity reduction rates from immediately after mixing (0 min) to 60 min were 37.0% for ET1 and 56.9% for ET2. These results suggest that the ether-based polycarboxylate superplasticizers are more stable in alkaline environments, allowing them to maintain their dispersing ability over a longer period. ET1 showed better performance in maintaining fluidity for 60 min.

In Section 3.1, it was confirmed that the range of molecular weight did not change and showed similar levels under both neutral and alkaline conditions for the ether-based polycarboxylate superplasticizers. This indicates that the ether-based polycarboxylate superplasticizers can perform well for mortar and concrete in a strongly alkaline environment. The stability of the ether bonds in these superplasticizers prevents molecular degradation, thereby enabling prolonged retention of fluidity. The fluidity test results for ET1 and ET2 showed that the ether-based polycarboxylate superplasticizers can improve fluidity in an alkaline environment and can extend the working time, providing more flexibility in handling and placing AAS pastes.

### 3.4. Setting Time

**Figure 5:**
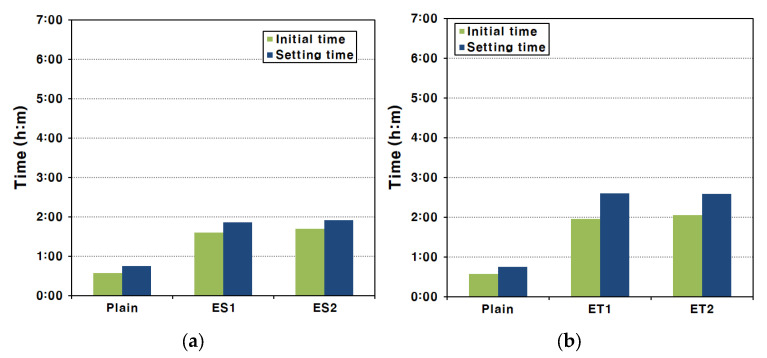
Initial and final setting times of AAS paste via the Vicat test method: (**a**) ES and (**b**) ET.

For AAS pastes that form in a strong alkaline environment, setting occurs rapidly and the reaction is much faster than the hydration reaction of Portland cement [39]. Therefore, AAS paste setting times must be measured in a period shorter than the existing setting measurement period in order to obtain an accurate setting time. This rapid setting is primarily driven by the fast ionization and dissolution of slag particles in the presence of alkali activators, leading to accelerated formation of hydration products. Figure 5 shows the setting time test results of the AAS pastes made with each type of polycarboxylate superplasticizer.

In the experimental results, the initial set occurred at 34 min and the final set at 45 min for the Plain mixture. These results indicate that the hardening process for AAS pastes is significantly faster compared to traditional Portland cement-based systems, where the setting typically takes much longer. This shows that hardening occurred rapidly compared with the initial setting criteria of ASTM C 150/C 150M cements [40]. The rapid setting can be attributed to the breakdown of the GGBFS film under strongly alkaline conditions, which facilitates the fast ionization and elution of ions from the interior of the GGBFS particles. Hence, the final set of AAS paste occurs approximately ten minutes after the initial set [39]. This short interval between the initial and final set is a characteristic of alkali-activated systems due to the rapid formation of hydration products.

Similar setting characteristics were observed for the AAS paste mixed with the ester-based polycarboxylate superplasticizers. The use of ester-based superplasticizers delayed the initial set slightly compared to the Plain mixture, but the time interval between the initial and final set was similar to that of the Plain mixture. This suggests that, while the ester-based polycarboxylate superplasticizers can influence the initial setting by improving fluidity, they do not significantly alter the overall setting process. For the AAS pastes made with the ether-based polycarboxylate superplasticizers, a more noticeable delay in the initial set was observed, and the time from the initial set to the final set also slightly increased. This delay in both the initial and final setting times for ether-based superplasticizers indicates a more pronounced effect on controlling the reaction kinetics in the early stages of setting. Even in this case, the time taken was short compared to that expected for the setting of conventional cement [13].

In the fluidity tests mentioned above, it was confirmed that the polycarboxylate superplasticizers improved the fluidity of AAS pastes. This increase in fluidity could slightly delay the time of initial set, which is the criterion for the loss of fluidity. The improved dispersion of slag particles by the superplasticizers likely delayed the formation of early hydration products, slightly extending the working time of the AAS paste. However, it was not possible to delay the final set of the AAS paste, which is the time when stiffness is formed due to bonding between the products of the hydration reaction. This is because, once the hydration reaction is fully initiated, the rate of hydration product formation is rapid and cannot be significantly influenced by the superplasticizers.

### 3.5. Rheology (VRR)

**Figure 6:**
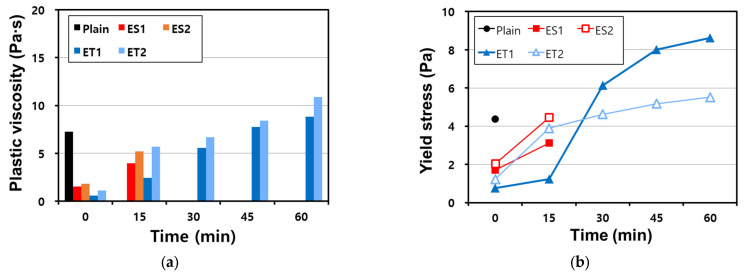
Results of the VRR test on AAS pastes with chemical admixtures: (**a**) plastic viscosity and (**b**) yield stress.

Figure 6 shows the plastic viscosity and yield stress of the AAS pastes made with the polycarboxylate superplasticizers through the VRR test. The plastic viscosity and yield stress can be measured using the equation for Bingham fluids, and they increase over time as the binder undergoes hydration reactions [35,36]. This increase is due to the progressive formation of hydration products, which leads to greater resistance to flow as the paste stiffens.

The results show that the VRR test could only be performed immediately after mixing (at 0 min) for the Plain mixture, and no further measurement could be performed owing to the loss of fluidity. At 0 min, the Plain mixture showed a plastic viscosity of 7.25 Pa⦁s and a yield stress of 4.37 Pa. This rapid loss of fluidity in the Plain sample reflects the highly reactive nature of AAS pastes, where the initial setting process is accelerated due to the strong alkaline environment. However, the polycarboxylate superplasticizers were able to decrease the plastic viscosity and yield stress compared with those of Plain mixture, suggesting that they improved the dispersing ability and delayed the stiffening of the paste.

The plastic viscosity and yield stress of the ether-based polycarboxylate superplasticizers were lower than those of the ester-based polycarboxylate superplasticizers. This difference can be attributed to the relative stability of the ether bonds compared to the ester bonds in an alkaline environment, as discussed in Section 3.1.

As shown in Table 2, there is no significant difference in molecular weight, which would affect the binder dispersion performance between the ether and ester polycarboxylate superplasticizers; all four polycarboxylate superplasticizers show similar viscosity.

However, the ester-based polycarboxylate superplasticizers exhibited higher plastic viscosity and yield stress because their molecular structures were compromised when mixed with the AAS paste. The strong alkaline environment leads to the hydrolysis of the ester bonds, resulting in a decrease in their dispersing efficiency.

The polycarboxylate superplasticizers have the function of maintaining fluidity, but the ether- and ester-based polycarboxylate superplasticizers showed a difference in performance. While the VRR test could not be conducted on the ester-based polycarboxylate superplasticizers after 30 min owing to the loss of fluidity and rheological characteristics, it could be conducted on the ether polycarboxylate superplasticizers even after 60 min because they maintained sufficient fluidity. This difference highlights the superior stability of the ether-based polycarboxylate superplasticizers in maintaining their molecular structure and dispersing function in the AAS paste.

There was a difference in rheological characteristics even between ET1 and ET2, which are both ether-based polycarboxylate superplasticizers. The plastic viscosity and yield stress of both superplasticizers increased over time as expected, but each exhibited a sudden increase in rheological resistance at different times. This occurred between 15 and 30 min for ET1 and between 0 and 15 min for ET2. These differences suggest that even minor variations in molecular composition or bonding strength can lead to significantly different behavior in a strongly alkaline environment. Further research is required to study the influence of molecular bonding on polycarboxylate superplasticizers in an alkaline environment. In particular, understanding how ether-based superplasticizers interact with the ionic species in the AAS system may help elucidate the mechanisms behind their superior performance in maintaining fluidity and controlling rheological properties.

### 3.6. Rheology (CCR) and UPV

**Figure 7:**
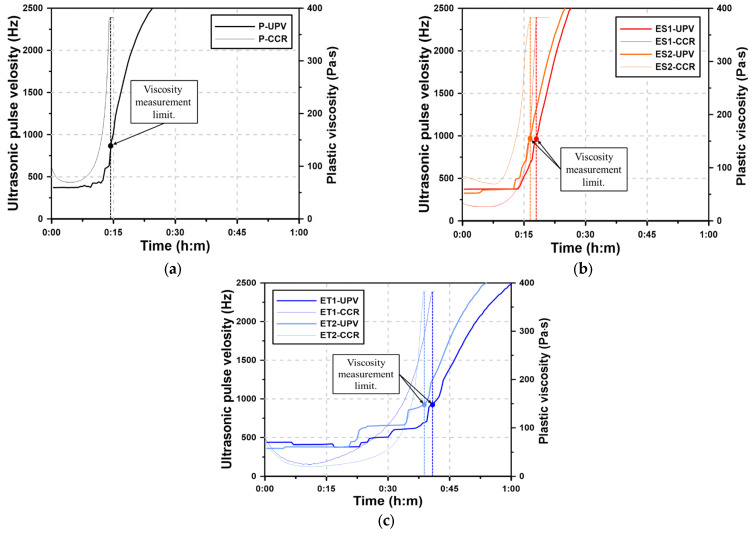
Results of the CCR test for AAS pastes with chemical admixtures: (**a**) Plain, (**b**) ES, and (**c**) ET.

Figure 7 shows the UPV curves for the AAS pastes and the plastic viscosity curves through the CCR test for one hour. In the CCR test, the spindle rotates at a constant speed, and plastic viscosity is measured as the AAS paste gradually loses fluidity. The AAS pastes harden over time due to the loss of fluidity, and the plastic viscosity curve can quantitatively represent the process by which the fluidity of the AAS paste decreases. This decrease in fluidity corresponds to the ongoing hydration reactions and the subsequent formation of hydration products. As the UPV increases due to the hardening of the AAS paste, the hardening process of the AAS paste can be predicted by analyzing the UPV curve over time [41,42]. In the CCR test, a spindle is rotated in the fluid paste, and measurements cannot be performed if the paste has lost its fluidity. This results in a loss of measurable rheological data as the paste transitions from a fluid to a plastic state.

The plastic viscosity curves of the AAS paste show the fluid-state viscosity immediately after mixing, but the rheological characteristics are lost after a short period of time due to the sharp increase in viscosity. This rapid increase in viscosity indicates the onset of the hydration reaction, where the formation of solid hydrates accelerates the transition from fluidity to rigidity. The time at which the plastic viscosity can no longer be measured using a rheology test device is referred to as the viscosity measurement limit (VML). The VML marks the point at which the AAS paste loses sufficient fluidity and transitions to a semi-solid state, indicating the beginning of hardening. The process by which the AAS paste loses fluidity is different from the process by which cement paste loses fluidity and reaches initial set. In general, cement paste slowly loses fluidity by consuming excess water, whereas AAS paste tends to harden rapidly in the fluid state rather than losing fluidity in a stepwise manner over time. This rapid transition is due to the highly reactive nature of alkali-activated materials, which form hydration products much more quickly than conventional cement.

However, in Section 3.5, the ether polycarboxylate superplasticizers showed measurable rheological characteristics until 60 min. This extended working time suggests that the ether-based superplasticizers are more effective in delaying the loss of fluidity compared to their ester-based counterparts. In the case of the VRR test, remixing was performed for 15 s before each measurement, and the plastic viscosity was recovered due to the subsequent disturbance of the paste. This remixing likely disrupted the initial network of hydration products, temporarily restoring the paste’s fluidity. Without the disturbance caused by remixing, fluidity was lost within one hour, as in the CCR test results.

The VML of the CCR test is related to the time of initial set when the AAS paste loses fluidity and changes to the plastic state. This correlation provides an insight into the early hardening behavior of AAS pastes. The Plain paste had the most rapid loss of fluidity with a VML of 14:20. The VMLs of ES1 and ES2 were 18:00 and 16:40, respectively, showing a difference of several minutes compared with Plain. This indicates that the ester-based superplasticizers provided a slight delay in the loss of fluidity, but did not significantly extend the working time. However, the VMLs of ET1 and ET2 were 40:40 and 38:40, respectively, indicating that fluidity could be maintained for more than 20 min longer compared with Plain. This demonstrates that ether-based polycarboxylate superplasticizers are more effective in maintaining fluidity and delaying the transition to a plastic state.

The use of UPV curve with respect to the VML make it possible to follow the hardening process of the AAS paste. The UPV maintains a constant low value while still in the fluid state, but it rapidly increases as the hydrates of the AAS binder are generated and interlock to form a solid. The increase in UPV corresponds to the formation of solid hydration products, which restrict the movement of ultrasonic waves, causing the velocity to rise sharply. Therefore, the onset of the increase in UPV indicates that the hardening of the AAS paste has begun. This is closely related to the VML, as both represent the point where the paste loses fluidity and transitions to a more rigid state. The UPV of the AAS paste was maintained at 300 to 500 Hz in the fluid state, and then the UPV rapidly increased regardless of the addition of the polycarboxylate superplasticizers. The Plain paste shows the earliest time of the increase in UPV followed by ES2, ES1, ET2, and ET1. This order suggests that the ether-based superplasticizers were the most effective in delaying the onset of hardening, as indicated by the slower rise in UPV.

The UPV results in Figure 7 show that the UPV where the VML occurs is in the 900–1,000 Hz range for the AAS paste. After VML, the UPV increased rapidly, indicating the occurrence of initial set time caused by the hydration reactions of AAS. The rapid rise in UPV after the VML point confirms that the paste transitioned to a rigid state, marking the onset of significant hydration product formation.

## 4. Conclusions

The molecular structures of both ester- and ether-based polycarboxylate superplasticizers in a strong alkaline environment were examined to improve the fluidity of AAS paste, and the fluidity and setting characteristics were analyzed during the first hour. The following conclusions could be drawn.

(1) The AAS paste rapidly loses fluidity due to the accelerated hydration reaction of the AAS binder, leading to premature hardening. This rapid setting behavior highlights the need for strategies to delay the hydration reaction to improve the fluidity and working time of the AAS paste. Potential solutions include adjusting the alkaline activator composition or incorporating chemical retarders.
(2) Ester-based polycarboxylate superplasticizers degrade in the highly alkaline environment of the AAS paste, resulting in poor fluidity retention. In contrast, ether-based polycarboxylate superplasticizers demonstrate better stability and significantly improve the fluidity of the AAS paste, allowing for extended working time. However, even with ether-based superplasticizers, the working time remains shorter compared to traditional OPC systems, indicating that additional measures, such as the use of retarders or fine-tuning the type and dosage of alkaline activators, are necessary to achieve optimal performance. It is important to consider the potential long-term effects of such adjustments on the mechanical strength and durability of the AAS paste, which warrant further investigation.
(3) As the AAS paste stiffens rapidly in the fluid state due to the fast hydration reaction, the conventional Vicat test may not adequately capture the initial setting behavior. Instead, the viscosity measurement limit (VML) and ultrasonic pulse velocity (UPV) tests provide a more accurate and quantitative means to evaluate both the fluidity and setting characteristics of the AAS paste. These methods allow for a better understanding of the early setting process, which is critical for optimizing the performance of AAS systems.
(4) Ether-based superplasticizers offer superior stability and fluidity retention in the highly alkaline environment of AAS systems compared to ester-based superplasticizers, but achieving optimal working time still requires additional measures such as the use of retarders or adjustments to the alkaline activator composition to balance short-term workability with long-term strength and durability.

In conclusion, ether-based superplasticizers show promise in improving the fluidity and workability of AAS pastes, but the rapid setting behavior characteristic of AAS systems still poses challenges [38]. Addressing these challenges requires a holistic approach that considers not only the choice of superplasticizers but also the broader mix design, including alkaline activator composition and the use of setting retarders. Future research should focus on optimizing these factors to balance both short-term workability and long-term strength and durability.

## Figures and Tables

**Table 1:** Physical properties and chemical composition of GGBFS.

Type	Density (g/cm^3^)	Blaine (cm^2^/g)	Particle Size Distribution (μm)				Chemical Composition (%)				
			d_m_	d_10_	d_50_	d_90_	CaO	SiO_2_	Al_2_O_3_	MgO	SO_3_
BFS	2.90	4253	14.2	1.4	10.2	33.2	44.0	33.7	13.8	5.2	1.2

**Table 2:** Properties of polycarbonate chemical admixture.

		Polyester Types		Polyether Types	
		ES1	ES2	ET1	ET2
pH		3.0 ± 1.0	3.0 ± 1.0	3.0 ± 1.0	3.0 ± 1.0
Viscosity (cPs)		300–600	300–600	300–600	300–600
Molecular mass		2200	2200	1200	2200
Sample Example		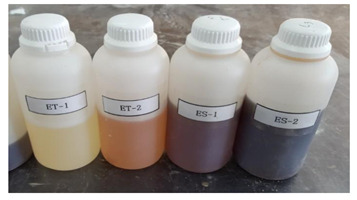			

**Table 3:** Mixture design of AAS pastes.

Liquid-to-Binder Ratio (l/b)	Liquid (g)	Binder (g)	Composition of Liquid			Chemical Admixture
			Alkali Activator (g)			
			Water	NaOH	Na_2_SiO_3_	
42%	420	1000	202.8	5.20	212.0	1%

**Table 4:** Paste viscometer specifications.

Model	Viscometer		Spindles(M-1)	
	Max (cPs)	Min (cPs)	Max (cPs)	Min (cPs)
DV-III Ultra	9360	156	200	100

**Table 5:** Properties of polycarbonate chemical admixtures.

		Plain	ES1	ES2	ET1	ET2
	
Second hydration heat inflection point		15:09	22:00	15:00	27:00	30:00
VML		14:20	18:00	16:40	38:40	40:40
Initial setting time		34:00	1:42	1:36	1:57	2:03

## Data Availability

The original contributions presented in the study are included in the article; further inquiries can be directed to the corresponding authors.
